# Heat shock factors in tomatoes: genome-wide identification, phylogenetic analysis and expression profiling under development and heat stress

**DOI:** 10.7717/peerj.1961

**Published:** 2016-05-10

**Authors:** Xuedong Yang, Weimin Zhu, Hui Zhang, Na Liu, Shoubo Tian

**Affiliations:** The Protected Horticulture Institute, Shanghai Academy of Agricultural Sciences, Shanghai, China

**Keywords:** Genome-wide analysis, HSF, Tomato, Expression profile

## Abstract

The HSF (heat shock factor) gene family contains highly conserved plant-specific transcription factors that play an important role in plant high-temperature stress responses. The present study aimed to characterize the HSF transcription factor genes in tomato (*Solanum lycopersicum*), which is an important vegetable crop worldwide and the model plant for fruit development studies. Twenty-six SlyHSF genes were identified in tomato, and the phylogenetic analysis showed the possible evolution profile of subgroups among in the plant kingdom. A new group O was identified that involved HSF genes in primitive plant species, like in the green algae, mosses and lycophytes. The gene structure and motifs of each SlyHSF were comprehensively analyzed. We identified orthologous, co-orthologous and paralogous HSF gene pairs in tomato, Arabidopsis and rice, and constructed a complex interaction network among these genes. The SlyHSF genes were expressed differentially in different species and at a higher level in mature fruits. The qPCR analysis was performed and showed SlyHSF genes greatly participate in plant heat tolerant pathways. Our comprehensive genome-wide analysis provided insights into the HSF gene family of tomatoes.

## Introduction

Global warming poses a threat to the production of various crops. Tomato (*Solanum lycopersicum*) is considered as an important and economic agricultural vegetable crop worldwide. Although tomatoes can survive in a wide range of climatic conditions, their vegetative and reproductive growth are severely impaired at heat stress conditions, resulting in reduced yield and fruit quality (Pressman, Peet & Pharr, 2002). Therefore, it is extremely important to improve the heat resistance of crops through molecular manipulation. Heat stress causes many heat-labile proteins to denature and harmful reactive oxygen species to increase in plant cells (Grover et al., 2013; Mittler, Finka & Goloubinoff, 2012). When faced with heat stress, the expression of heat-shock genes increases rapidly, leading to the rapid accumulation of heat-shock proteins (HSPs). Expression of HSPs is mainly regulated by heat shock transcription factors (HSFs) on a transcriptional level, and they play a critical role in high-temperature stress responses (Lin et al., 2011).

Plant HSF genes, first identified from tomato, have been isolated from various species (Aranda et al., 1999; Czarnecka-Verner et al., 1995; Hubel & Schoffl, 1994; Scharf et al., 1990). In contrast to other eukaryotes such as *Drosophila melanogaster*, *Caenorhabditis elegans* and yeast with a single HSF gene in the genome, plants possess a large family of HSFs. For example, a previous report identified *Arabidopsis* and rice (*Oryza sativa*) possessed 21 and 25 HSF genes respectively (Guo et al., 2008). Like many other transcription factors, the HSF family has a conserved modular structure containing highly conserved domains (Doring et al., 2000; Treuter et al., 1993). The conserved structure elements include an N-terminal DNA binding domain (DBD), an adjacent domain with heptad hydrophobic repeats (HR-A/B) involved in oligomerization and the nuclear localization signal domain (NLS) (Guo et al., 2008). In addition, some HSFs have a C-terminal activation domain (CTAD) and a nuclear export signal (NES) domain (Kotak et al., 2004). Based on their flexible linkers between the A and B parts of the HR-A/B regions and the sequence regions between the DBD and HR-A/B regions, plant HSFs can be classified into three types (class A, B, and C) (Nover et al., 2001; Nover et al., 1996). HSFs act through a highly conserved heat shock element (HSE) containing motifs in alternating orientations in the promoters (Schoffl, Prandl & Reindl, 1998). Class A HSFs are involved in transcriptional activation and environmental stress responses (Shim et al., 2009), while class B HSFs act as repressors of gene expression (Ikeda, Mitsuda & Ohme-Takagi, 2011; Zhu et al., 2012). Previous research showed that HSFB1 in *Arabidopsis* acts as a repressor, while in tomato, it functions as a transcription co-activator with class A HSFs (Ikeda, Mitsuda & Ohme-Takagi, 2011; Zhu et al., 2012).

The HSF gene family has been thoroughly characterized in many species, including *Arabidopsis*, Chinese cabbage, rice, maize, *Triticum aestivum*, pepper and grasses (Guo et al., 2008; Lin et al., 2011; Nover et al., 2001; Song et al., 2014; Xue et al., 2014; Yang et al., 2014). Although tomato HSFs have been identified and classified (Doring et al., 2000; Heerklotz et al., 2001; Scharf et al., 2012; Scharf et al., 1990), but only the identification was done in that paper. This study is the first comprehensive report of tomato HSFs, the chemical characteristics of the proteins have been obtained, and compared with other organisms. A phylogenetic tree using representative species including green alga, moss, lycophyte, gymnosperm, monocot and eudicots has been constructed in this study, in order to study the HSF classification and evolution across the whole plant kingdom. Furthermore, the expression patterns of all tomato HSF genes in different tissues and after treated in high temperate tress have been characterized. The results of this work provide a foundation to better understand the functional and evolutionary history of the HSF gene family in Solanaceae plants.

## Materials and Methods

### Identification and characteristics of tomato HSF genes

The genome, gene and protein sequences of tomato were downloaded from the Sol Genomics Network database (http://solgenomics.net, ITAG 2.40). The HSF-domain search model accession PF00447.12 in Pfam database was used to search against all 34725 tomato genes using HMMER, with a strict cut-off E-value of 10^−5^. The positions of each HSF-coding gene on chromosomes were obtained according to Tomato Genome Annotation ITAG 2.40. Protparam program (http://web.expasy.org/protparam/) was employed to calculate or predict the chemical characteristics of tomato HSF proteins, including the molecular formula of the protein, number of amino acids per protein, molecular weight, estimated theoretical pI, instability index, aliphatic index and GRAVY (Grand Average of Hydropahicity).

### Phylogenetic analyses construction

HSF proteins for phylogenetic analyses were gathered from eight plant species. The proteins of *Picea abies* was downloaded from http://congenie.org (Nystedt et al., 2013). The six other species, including *Chlamydomonas reinhardtii, Physcomitrella paten, Selaginella moellendorffii, Arabidopsis thaliana*, *Vitis vinifera* and *Oryza sativa*, were downloaded from Phytozome database (v10) (Goodstein et al., 2012). The HSF proteins with their conserved domains were also screened by the HMMER software. Only the longest transcript was used if alternative spliced isoforms existed. After multiple sequence alignment of the HSF domains using ClustalX2 software with default settings, MEGA (version 6.06) was used to construct maximum-parsimony phylogenetic trees with 2,000 bootstrap replicates.

### Gene structure and motif analysis

The Gene Structure Display Server tool (http://gsds1.cbi.pku.edu.cn/) was used to analyze the exon-intron structures. The gene structures of tomato HSF were drawn using Photoshop software, including a clustering of all HSF genes in accordance with previously mentioned conserved protein domains. Besides the exon and intron regions, the upstream and downstream UTR regions were also reported to show possible structures of entirely expressed mRNA. Intron phases were classified based on their positions relative to the reading frame of the translated proteins: phase 0 (located between two codons), phase 1 (splitting codons between the first and second nucleotides) or phase 2 (splitting codons between the second and third nucleotides) (Long, Rosenberg & Gilbert, 1995). The software MEME (http://meme.nbcr.net/meme/) (Bailey & Elkan, 1994) was used to search for motifs in all 26 HSF genes; the number of motifs that MEME should find was set to 15 in this study. The length of motifs that MEME searched was in a window of 6 to 50 bp.

**Table 1:** Genomic characteristics of SlyHSF genes in tomato.

ID	Name	Chromosome	Strand	Start	Stop	No. Gene bases (bp)	No. cDNA bases (bp)	No. CDS bases (bp)	No. Amino acids (aa)
SlyHSF-01	Solyc11g064990.1	SL2.40ch11	−	47389718	47391840	2,123	756	756	251
SlyHSF-02	Solyc08g005170.2	SL2.40ch08	−	111412	116839	5,428	1,949	1,584	527
SlyHSF-03	Solyc03g026020.2	SL2.40ch03	+	7810489	7812280	1,792	1,594	1,017	338
SlyHSF-04	Solyc03g097120.2	SL2.40ch03	−	52901766	52904929	3,164	1,874	1,476	491
SlyHSF-05	Solyc02g090820.2	SL2.40ch02	−	46880125	46883382	3,258	1,611	906	301
SlyHSF-06	Solyc09g065660.2	SL2.40ch09	+	59473864	59475995	2,132	1,405	1,119	372
SlyHSF-07	Solyc04g078770.2	SL2.40ch04	+	61036586	61037903	1,318	1,230	1,083	360
SlyHSF-08	Solyc06g072750.2	SL2.40ch06	+	41255352	41258348	2,997	1,613	1,449	482
SlyHSF-09	Solyc12g098520.1	SL2.40ch12	+	48549454	48552229	2,776	1,437	1,437	478
SlyHSF-10	Solyc08g080540.2	SL2.40ch08	−	60985869	60987278	1,410	1,329	978	325
SlyHSF-11	Solyc04g016000.2	SL2.40ch04	−	6594909	6598451	3,543	1,320	714	237
SlyHSF-12	Solyc12g007070.1	SL2.40ch11	−	50695058	50703526	8,469	1,110	1,110	369
SlyHSF-13	Solyc09g082670.2	SL2.40ch09	+	63781968	63784228	2,261	1,458	1,071	356
SlyHSF-14	Solyc06g053960.2	SL2.40ch06	−	33333411	33336335	2,925	892	429	142
SlyHSF-15	Solyc08g076590.2	SL2.40ch08	−	57710679	57714096	3,418	1,806	1,473	490
SlyHSF-16	Solyc09g059520.2	SL2.40ch09	−	50372011	50379351	7,341	1,551	1,170	389
SlyHSF-17	Solyc02g072000.2	SL2.40ch02	+	35903150	35904957	1,808	1,712	1,227	408
SlyHSF-18	Solyc08g062960.2	SL2.40ch08	−	49589145	49591151	2,007	1,215	1,056	351
SlyHSF-19	Solyc10g079380.1	SL2.40ch10	−	60125137	60126209	1,073	768	768	255
SlyHSF-20	Solyc03g006000.2	SL2.40ch03	+	678142	679948	1,807	1,693	1,206	401
SlyHSF-21	Solyc07g055710.2	SL2.40ch07	−	60972388	60973952	1,565	1,477	1,167	388
SlyHSF-22	Solyc07g040680.2	SL2.40ch07	+	46702761	46704429	1,669	1,435	1,071	356
SlyHSF-23	Solyc02g078340.2	SL2.40ch02	+	37643661	37646059	2,399	666	618	205
SlyHSF-24	Solyc09g009100.2	SL2.40ch09	−	2445341	2448016	2,676	1,959	1,530	509
SlyHSF-25	Solyc02g079180.1	SL2.40ch02	+	38360060	38365669	5,610	1,245	1,245	414
SlyHSF-26	Solyc02g072060.1	SL2.40ch02	+	35912808	35914648	1,841	1,020	1,020	339

**Figure 1:**
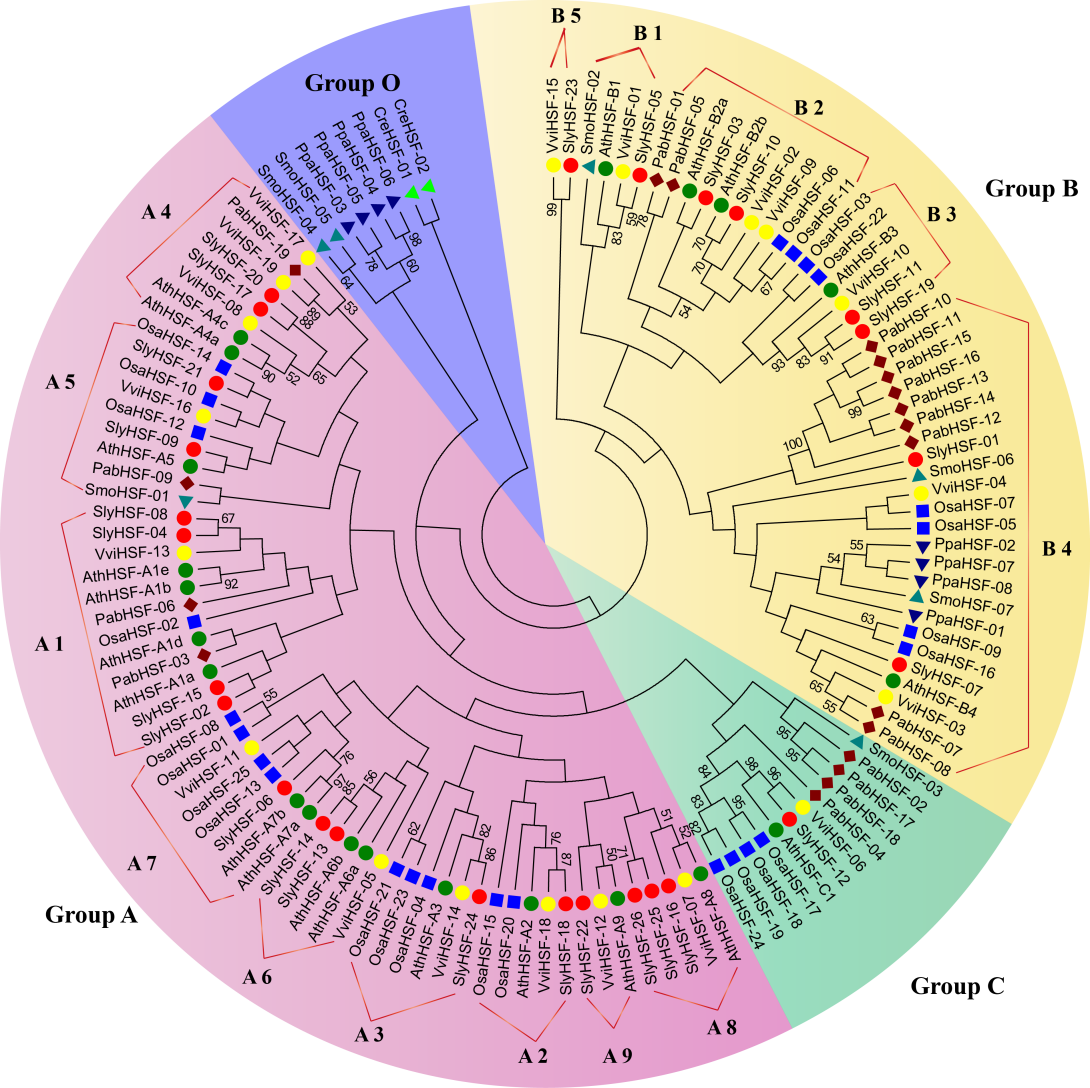
Phylogenetic tree of eight plants constructed based on amino acid sequences of HSF domains using the maximum-parsimony method. The different species can be distinguished by different shapes and colors.

### Identification of orthologous and paralogous genes

The orthologous, co-orthologous and paralogous genes among tomato, *Arabidopsis* and rice were searched using OrthoMCL (version 2.0.3) with the entire protein sequence of HSF. The default parameter E-value was 1e^−5^ for BLASTP in all vs all sequences alignment. The OrthoMCL software was used to gather the orthologous and paralogous relationships and the result was displayed using the Circos software (http://circos.ca/) (Krzywinski et al., 2009).

### HSF gene expression analysis in tomato tissues

The gene expression data was downloaded from the Tomato Funtional Genomics Database (http://ted.bti.cornell.edu/cgi-bin/TFGD/digital/experiment.cgi?ID=D004), including RNA-seq data from leaves, roots, flower buds, fully opened flowers, and 1 cm, 2 cm, 3 cm, mature green, breaker, and breaker + 10d fruits of tomato cultivar Heinz 1706, and leaves, immature green, breaker, and breaker + 5d fruits of *Solanum pimpinellifolium* LA 1589 (Tomato Genome, 2012). The SlyHSF gene expression profile from each sample was analyzed via the HemI program (http://hemi.biocuckoo.org/) with the average hierarchical clustering method.

**Figure 2:**
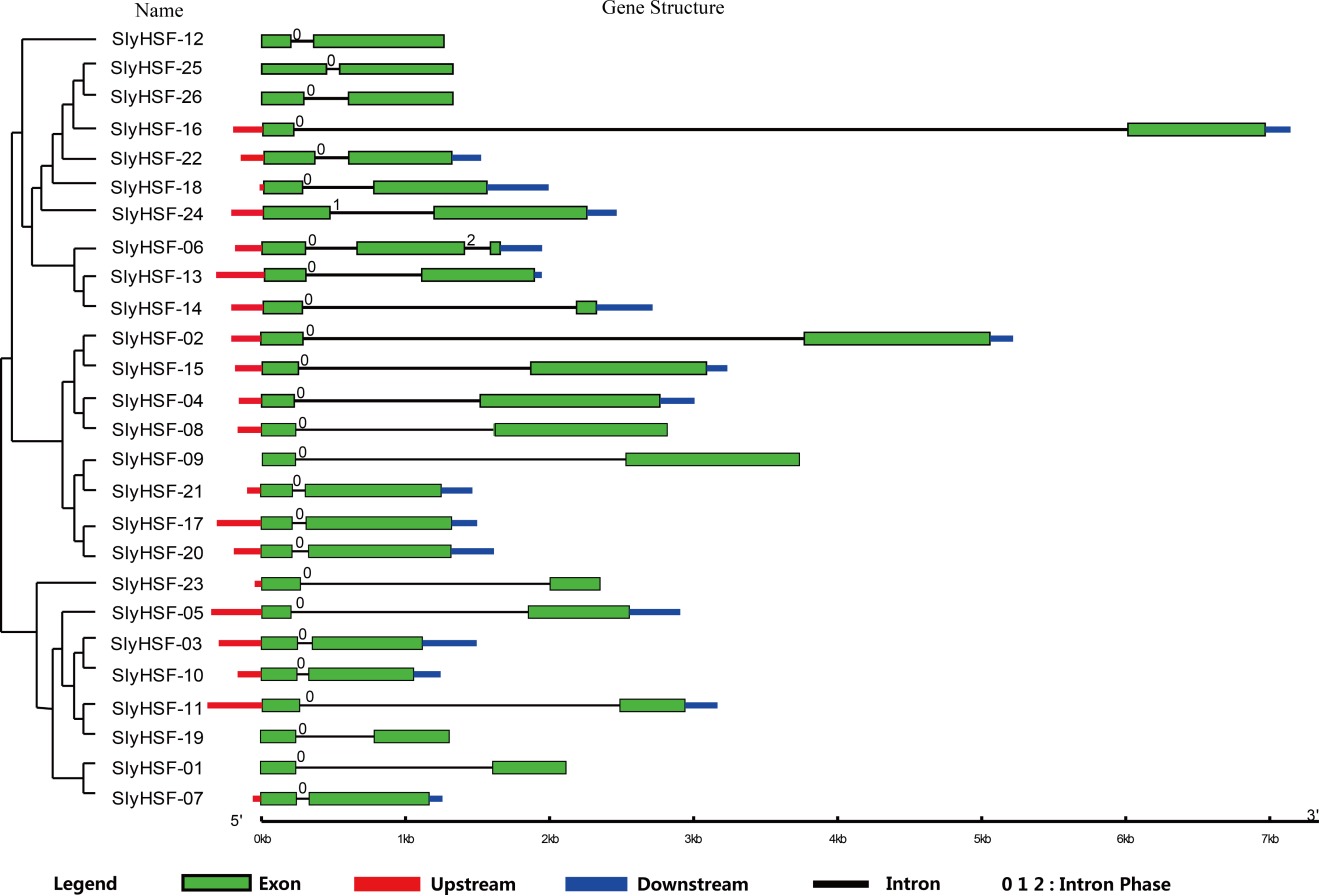
Gene structure of SlyHSF genes. Green boxes indicate the exon regions, while black, red and blue lines indicate introns, upstream and downstream UTR regions, respectively. The lengths of the boxes and lines were scaled based on the length of the genes.

### HSF gene expression analysis under heat treatment

Seeds from the tomato cultivar Heinz 1706 were germinated and grown in a greenhouse at 24 °C with a photoperiod of 14 h light and 10 h dark. For the heat stress treatment, uniform-sized seedlings were transferred to a growth chamber at 38 °C when they developed five fully opened leaves. The third leaves of the seedlings were taken after 0, 1, 2, 6, 12 and 24 h of heat stress treatment, frozen immediately in liquid nitrogen, and stored at −80 °C until RNA isolation. Total RNA was isolated from tomato leaves using a TaKaRa MiniBEST Plant RNA Extraction Kit (9769), according to the manufacturer’s instructions. The RNA was used to synthesize first-strand cDNA using M-MLV reverse transcriptase (TakaRa, Japan). The SYBR Premix Ex Taq II reagent (Takara, Japan) with SYBR Green I as the fluorescent dye was used for the qPCR using an ABI 7300 real-time PCR system (Applied Biosystems, Foster City, CA, USA). Each reaction contains 10 µL 2× SYBR Premix Ex Taq II Reagent, 1.0 µL cDNA sample, and 2 µL gene-specific primer in a final volume of 20 µL, 500 nM gene-specific primer in a final volume of 20 µL. RNA expression levels relative to the Actin2 (AB199316) gene were calculated according to a previous workflow (Pfaffl, 2001). Three replicates of each cDNA sample were performed for qRT-PCR analysis.

## Results and Discussion

### Identification and classification of HSF genes in tomato

A total of 26 HSF genes were identified in tomato based on a HSF domain search of all reference gene models (Table 1, Table S1). These genes were named after ‘SlyHSF’ with a serial number, sorted by E-value. According to tomato genome ITAG 2.40 Annotation Dataset, these genes were distributed unevenly on tomato chromosomes. Five SlyHSF genes were located on chromosome 2; four on chromosomes 8 and 9; three on chromosome 3; two on chromosomes 4, 6, 7 and 11; one each on chromosomes 11 and 12. The average length of the cDNA (exon + intron, ∼1,389 bp) for SlyHSF genes is longer than that of all tomato and cDNA. The coding sequence sizes for SlyHSF ranged from 429 bp (SlyHSF-14) to 1,584 bp (SlyHSF-02). The average number of amino acids in each SlyHSF was ∼366.7 bp, which is comparable to pepper (∼366.2 bp) and *Arabidopsis thaliana* (∼368.0 bp). As the oldest organism among plant kingdom, there were only two HSF proteins in algae, and both were much longer than found in other organisms. In contrast, the average length of HSF proteins in *S. moellendorffii* was only around 175 bp, which was shorter than other selected organisms.

Physical and chemical characteristics of SlyHSF proteins were analyzed and summarized in Table S2. The molecular weights were from 16.6 kDa to 57.5 kDa. The predicted isoelectric points of SlyHSF were divergent, ranging from 4.68 to 9.66. The instability index of all the proteins fell into a narrow range, from 31.89 to 68.07. The SlyHSF-05 and SlyHSF-14 protein molecule were predicted to be stable while others were unstable. All of the GRAVY scores were lower than 0, indicating that all SlyHSF proteins were hydrophilic.

### Phylogenetic relationship of SlyHSF proteins

For phylogenetic analysis, we selected eight other well-studied and representative plant species, including green algae (*Chlamydomonas reinhardtii*), moss (*Physcomitrella patens*), lycophyte (*Selaginella moellendorffii*), a gymnosperm (*Picea abies*), a monocot (rice, *Oryza sativa*) as well as three eudicots (*Arabidopsis thaliana*, grape and tomato) (Table S3). All plant HSFs were classified into four groups. Among them, Group A and Group C HSFs have an extended HR-A/B region, while class B HSFs have no insert sequences (Nover et al., 2001). According to the phylogenetic analysis, the 127 HSF proteins could be classified into Group A, B, C and O (Fig. 1). We named the new group O, which only contained two algae HSF proteins, four moss HSF proteins and two *S. moellendorffii* HSF proteins, because they shared less similarity with members in Group A , B and C. Among the 26 tomato HSF proteins, 17 members belonged to Group A and eight members belonged to Group B, while only one HSF was classified into Group C. The subgroups in Group B and Group A were defined according to a previous study of *Arabidopsis* HSF proteins (Guo et al., 2008). Since the phylogenetic tree was constructed using representative plant species, the divergence and emergence of HSF proteins in each subgroup could be predicted. After evolution from members in Group O, the HSF proteins of *Physcomitrella patens* existed only in subgroup B4, indicating this was the second oldest subgroup. Subsequently, the HSF proteins in subgroup A5, B1 and Group C emerged, first in lycopods. Additionally, the phylogenetic analysis revealed that tomato HSF members were more related closely to those from eudicots than to those from monocots and then other primitive plants (Fig. 1).

**Figure 3:**
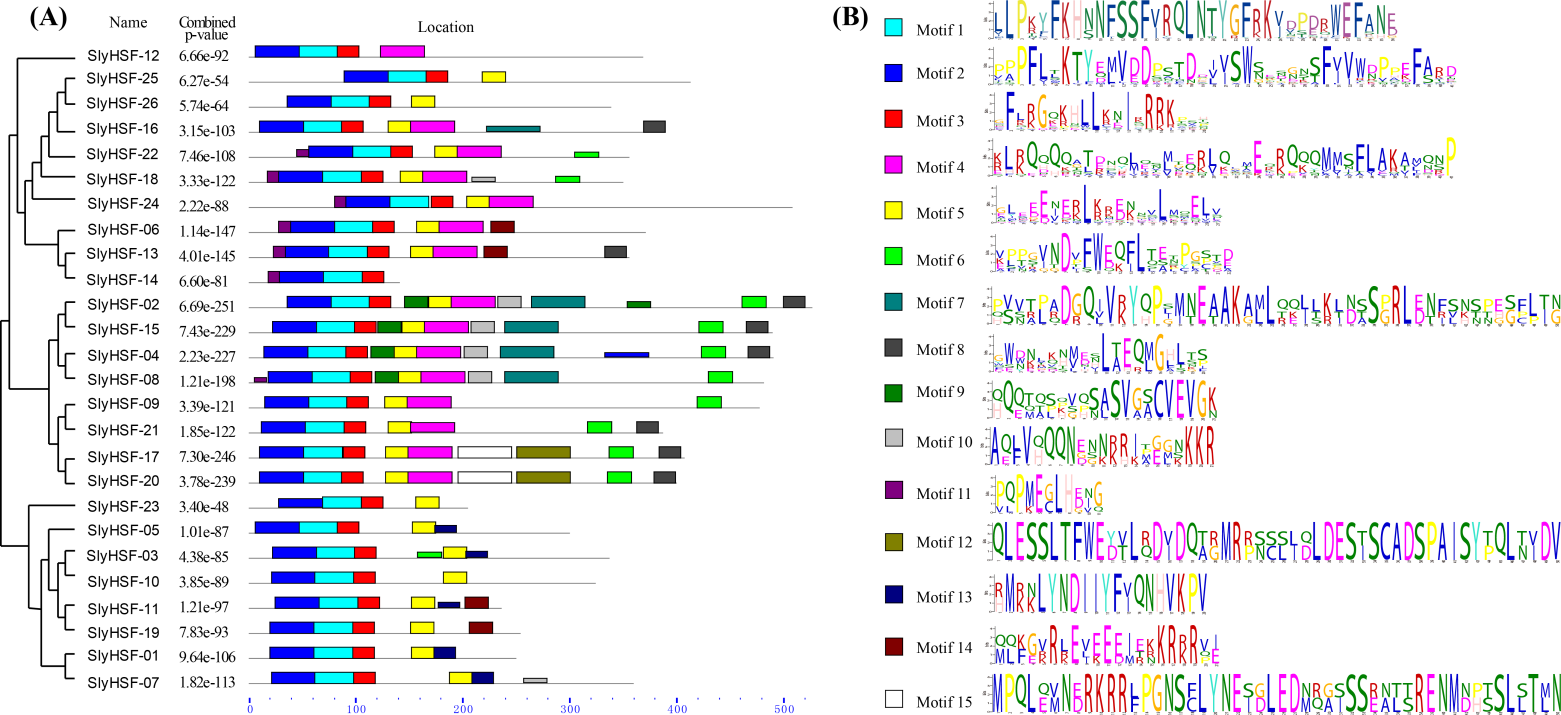
Conserved motifs arrangement in the SlyHSF genes. (A) The phylogenetic tree and motifs located on each gene with relative combined *P*-values. (B) Amino acid sequences of each motif. The font size represents the frequency of the respective amino acid.

The N-proximal regions of HSF genes and the neighbor-joining method were employed in phylogenetic tree construction in a previous study, resulting an ambiguous phylogenetic tree. In that study, subgroups A4 and A5 were arranged in close association with their related A subgroups, rather than among B subgroups as depicted in the previous study (Guo et al., 2015). In the present study, the conserved HSF domain regions aligned with Pfam model were used to construct a better phylogenetic tree using the maximum-parsimony method. However, the phylogenic clustering of rice HSF genes was still not entirely consistent with subgroup classification among genome-wide identification studies.

**Figure 4:**
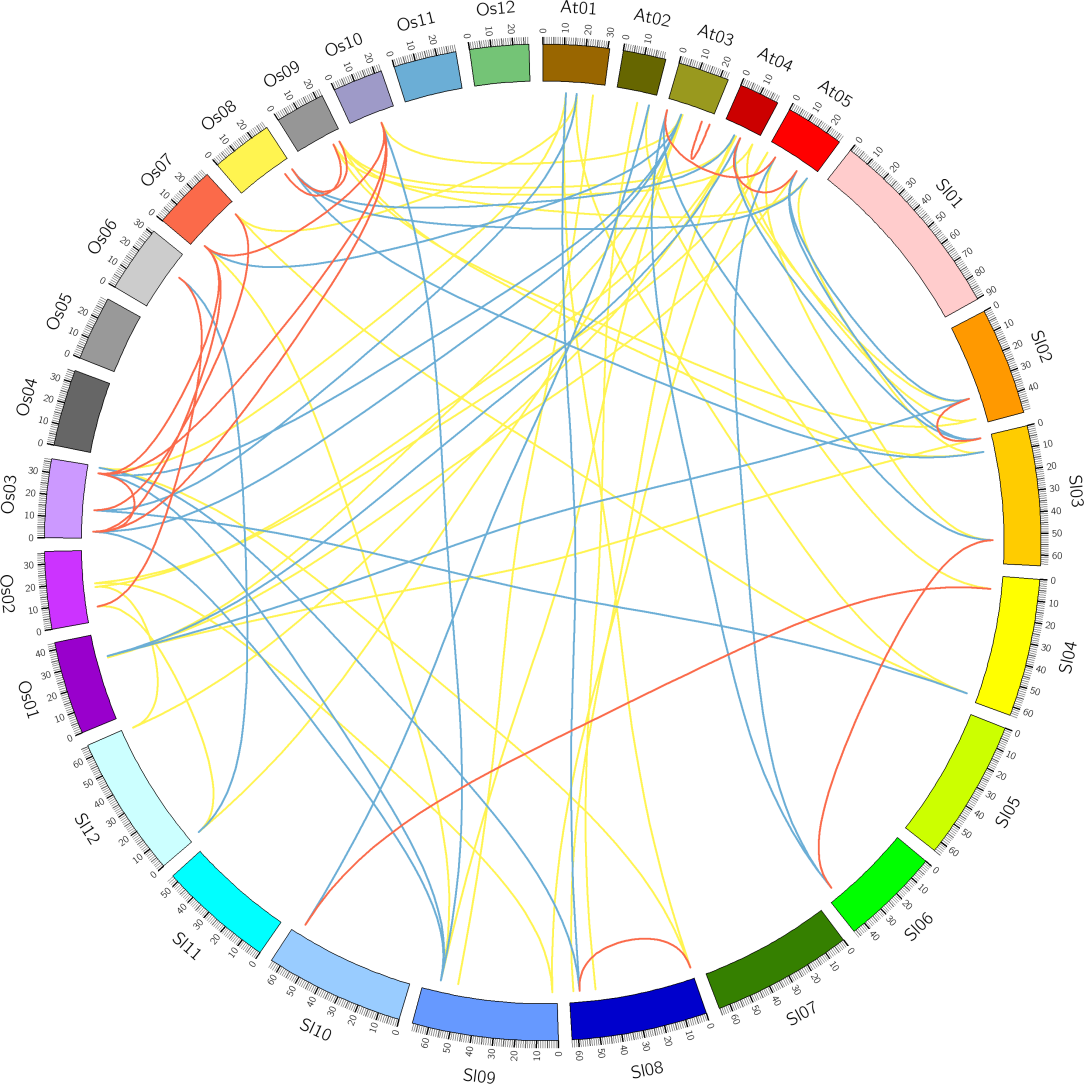
Comparative analysis of synteny of HSF genes in tomato, *Arabidopsis* and rice. Red, yellow and blue lines indicate paralogous, orthologous and co-orthologous gene pair relationships, respectively.

### Gene structure and motif analysis

To compare the 26 tomato HSF genes, their exon-intron structures were drawn (Fig. 2). All HSF genes were found to contain two exons and one intron, except for SlyHSF-06 which contained three exons. The intron phases were 0, except for phase 1 in SlyHSF24 and phase 2 in SlyHSF06. Although tomato HSF genes shared similar intron number and intron phase, the intron length differed in the groups. In subgroup A8, the intron in SlyHSF16 was much longer than SlyHSF25 or SlyHSF26. In addition, the lengths of introns fell in a wider range than that of exons, even in a subgroup.

We searched for motifs to analyze the conserved features of SlyHSF proteins using MEME (Fig. 3) Motifs 1, 2 and 3 were found in all tomato HSF members, while motif 5 was absent only in SlyHSF12 and SlyHSF14. As a counterpart of SlyHSF13, SlyHSF14 may have gone through a duplication during which the latter half was lost, resulting in shorter coding regions and fewer motifs. The similarity of motifs in subgroup A1 and subgroup A5 showed the close relationship between these two groups.

**Figure 5:**
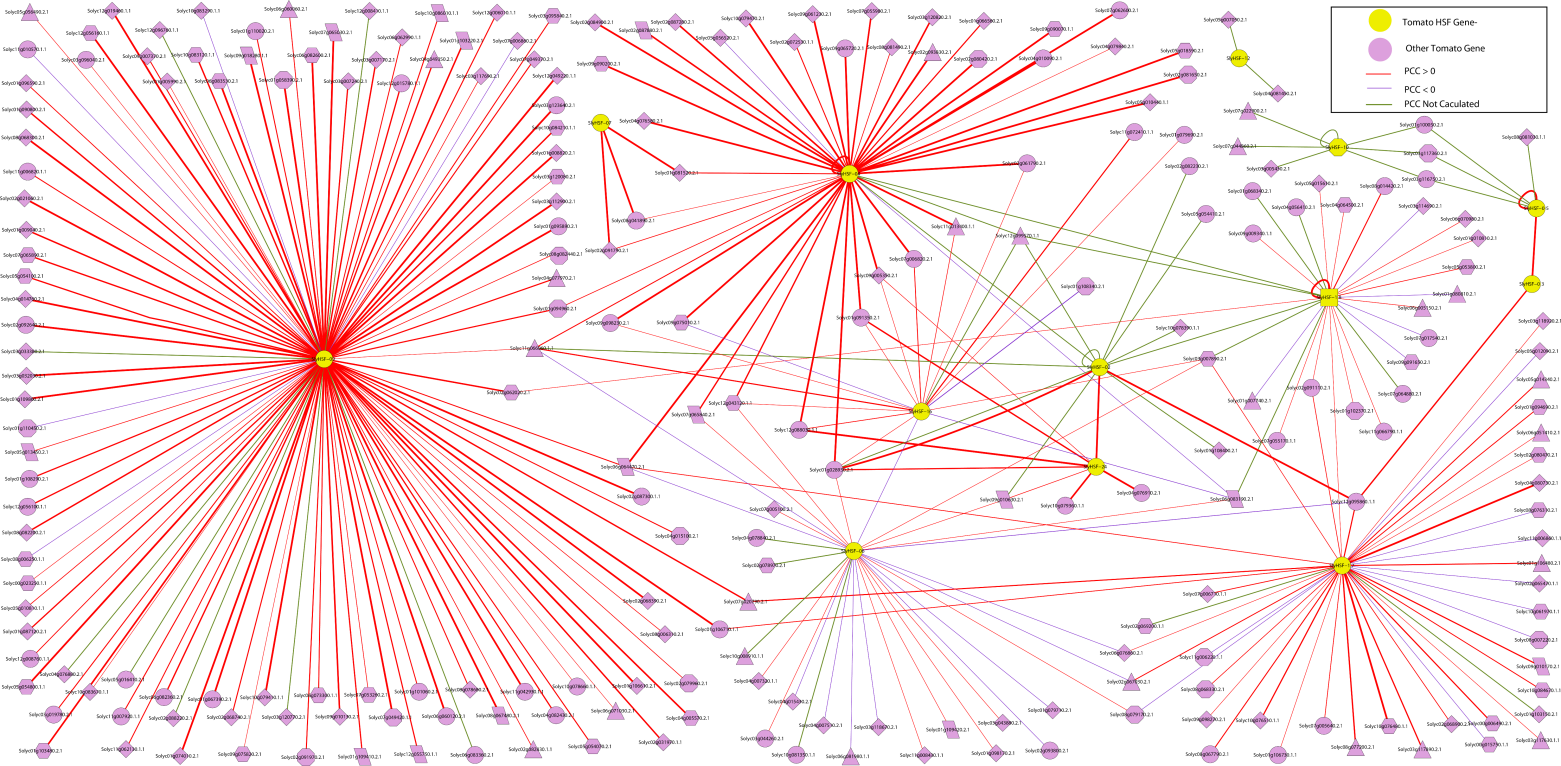
The interaction network of SlyHSF genes according to the networks in *Arabidopsis*. The shape of gene boxes illustrated the location of this gene. The ellipse, hexagon, V, parallelogram, triangle, octagon and diamond represented other tomato genes in plasma, membrane, vacuole, plastid and peroxisome, cytosol, others and unclear, respectively. Red, purple and green lines indicated that the Pearson correlation coefficient (PCC) index were greater than 0, less than 0 and not calculated respectively. The boldness of the line indicated the PCC value between genes.

**Figure 6:**
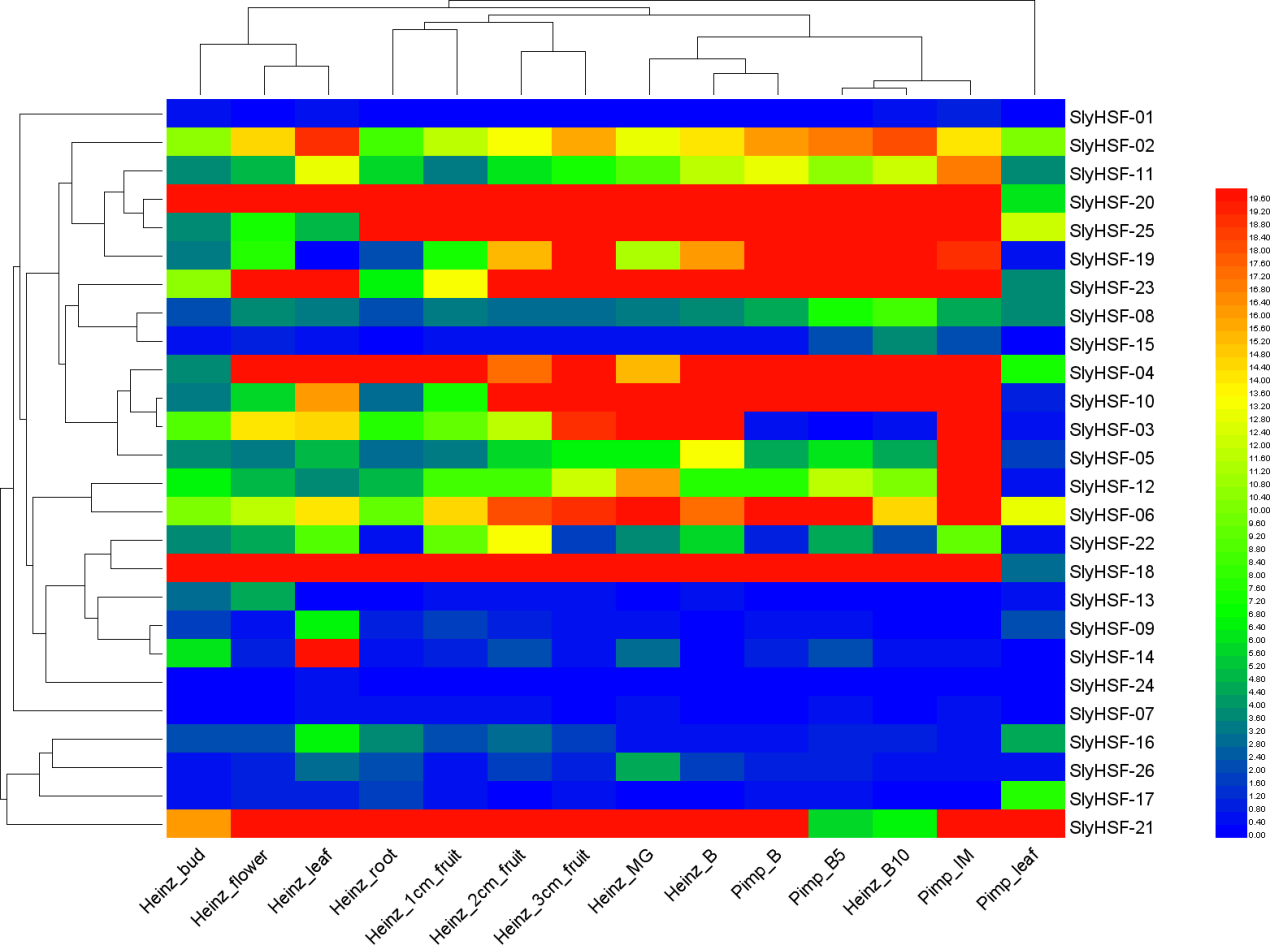
Heat map representation and hierarchical clustering of tomato SlyHSF genes in fourteen samples from root, leaf, bud, flower and fruits in several development stage. Heinz and Pimp represent the cultivated tomato Heinz and related wild species *Solanum pimpinellifoliumd*. IM, B, B5, B10 and M represent immature, breaker, 5 days after breaker, 10 days after breaker and mature fruit.

**Figure 7:**
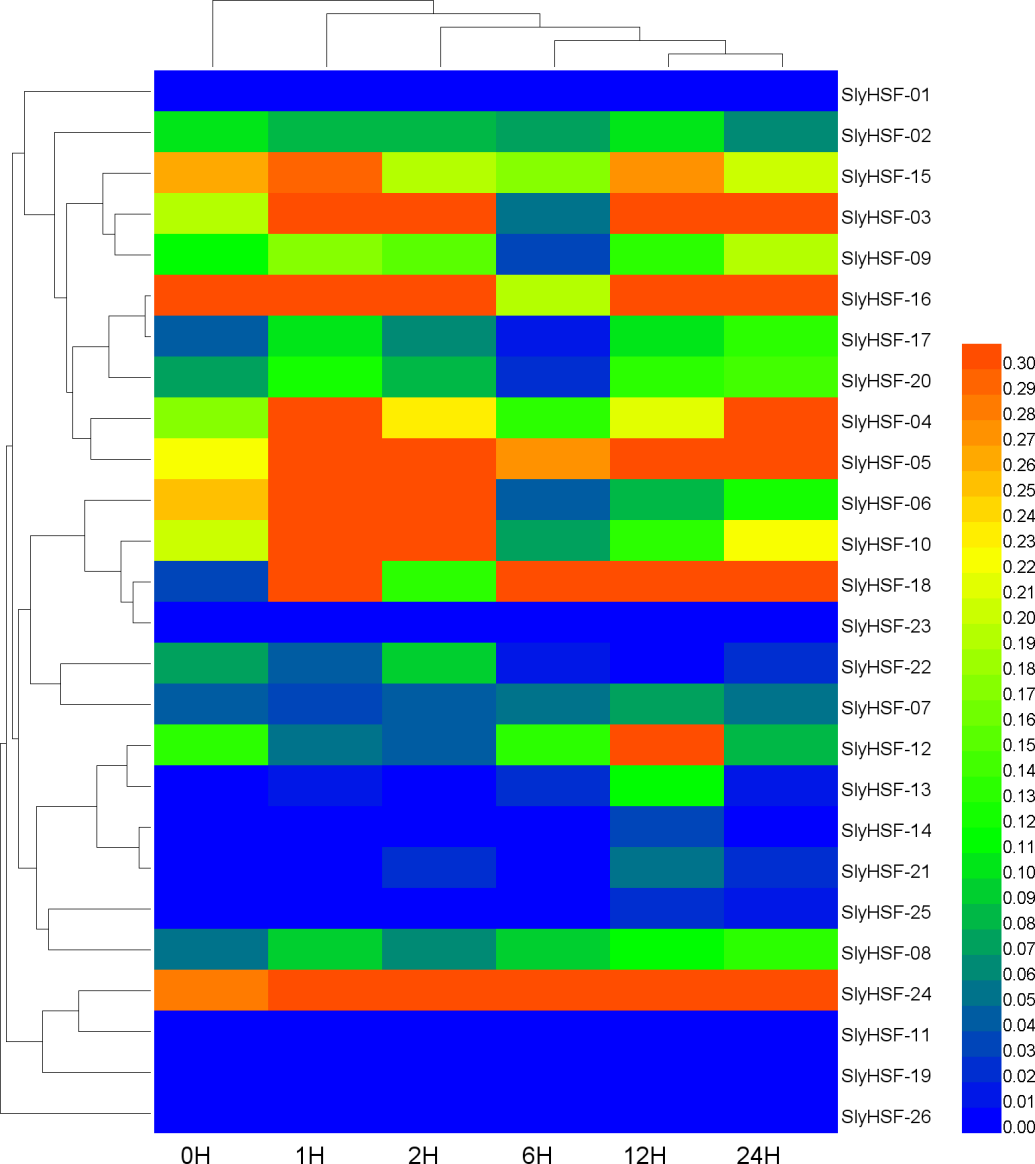
Hierarchical clustering and heat map representation of HSF genes after heat stress treatment. A qPCR experiment was performed to generate these expression profile data. The RNA level is expressed relative to the tomato actin gene expression level as 2^−ΔΔ*CT*^.

### Identification of orthologous and paralogous HSF genes in plants

Comparative analysis was performed to identify the orthologous and paralogous gene pairs. There were three, ten and four in-paralogous HSF gene pairs among *Arabidopsis*, rice and tomato respectively. The orthologous SlyHSF-AthHSF gene pairs (14 pairs) were identified as twice as SlyHSF-OsaHSF gene pairs (7 pairs), while ten AthHSF-OsaHSF gene pairs were found. Eight co-orthologous gene pairs (SlyHSF-AthHSF and SlyHSF-OsaHSF) were found (Table S4, Fig. 4).

An interaction network of SlyHSF genes was constructed to improve our understanding of the genome-wide regulation network (Fig. 5). First, an *Arabidopsis* HSF gene interaction network was constructed in the *Arabidopsis* Interactions Viewer (http://bar.utoronto.ca/interactions/cgi-bin/arabidopsis_interactions_viewer.cgi). Then, the *Arabidopsis* genes were replaced by their counterpart in tomato, using the orthologous and co-orthologous gene pairs. The PCC (Pearson correlation coefficients) of 38 gene pairs was less than zero, whereas that of 227 gene pairs was more than zero, which revealed SlyHSF proteins mainly have a positive interaction with other proteins in tomato. Fifty-three gene pairs were not included in the calculations, thus many regulation patterns remain unknown.

The interaction network showed that the number of proteins regulated by each SlyHSF gene was significantly different (Fig. 5). For instance, SlyHSF-09 had the most complex interaction network, with 114 proteins, suggesting its importance in transcriptional-level regulation. Furthermore, SlyHSF-02, SlyHSF-04, SlyHSF-06, SlyHSF-16, SlyHSF-17, SlyHSF-18 were found to have more than 10 interaction relationships, while SlyHSF-03, SlyHSF-05, SlyHSF-07, SlyHSF-10 and SlyHSF-12 had between 1 to 10 interaction relationships. Interestingly, the gene pairs including SlyHSF-02/04, SlyHSF-02/18, SlyHSF-02/24, SlyHSF-03/05, SlyHSF-04/18, SlyHSF-06/16 and SlyHSF-17/18 showed that interactions also existed among tomato HSF genes.

### Expression pattern of SlyHSF genes in different tissues

To increase our understanding of the expression profiles of the SlyHSF genes in different tissues, we searched gene expression values for each SlyHSF gene using reported RNA-seq data of *Solanum lycopersicum* and its closest wild relative *Solanum pimpinellifolium* (Table S5, Fig. 6). According to the FPKM values, at least 25 SlyHSF genes were expressed in at least one tissue. (It is possible that the expression in the root of cultivar Heinz was false positive). In these two species, the expression of HSF genes in *Solanum lycopersicum* Heinz were higher than in *Solanum pimpinellifolium*, which supported that the cultivated tomato was more heat-tolerant than *Solanum pimpinellifolium*. In these two species, the expression profile of SlyHSF genes in leaf, root, bud and flower tissues were in a group according to the clustering, while small fruit (1 cm, 2 cm and 3 cm) and bigger fruit (immature, breaker, breaker + 5d, breaker + 10d) could be arranged into another two groups, indicating that HSF genes were enriched in tomato fruit development process. In non-stress conditions, HSFA1a is reported to be repressed by association with HSP90 and HSP70 (Liu, Liao & Charng, 2011), therefore the SlyHSF-15 which share similar domains with AthHSFA1a were found to have lower expression than others.

### Expression pattern of SlyHSF genes under heat stress treatment

Since HSF genes were found to participate in heat shock related pathways, quantitative real-time PCR analysis was performed to systematically detect HSF gene expression in tomato. The non-conserved regions were used for primer design to ensure the specificity of PCR amplification (Table S6). Under heat stress treatment, the expression of most SlyHSF genes increased dramatically. The SlyHSF-05/07/13/18/20/23/24 genes were expressed more in all samples after heat stress treatment. The expression of the SlyHSF-18 gene increased over 150 times in 1 h compared with the control, suggesting that it was a very sensitive response acceptor that responded strongly. After that, the expression of SlyHSF-18 decreased 3.7 to 22.2 times in the following 23 h. The expression profile of SlyHSF-23 fell in a similar model that after peaking at 1 h (around 79-fold), then the expression decreased gradually to 15 times than the control (Table S7, Fig. 7). It has been shown that HSFA1a (termed SlyHSF-02 in this study) in tomato is a master regulator for triggering the heat response and can result in acquired thermotolerance (Mishra et al., 2002), although HSFA1a regulation was not significant in this study.

### Duplication among SlyHSF genes

After gene duplication, some regions of proteins disappeared. In Group A8, there were three tomato HSF proteins that share ancestory with *Arabidopsis thaliana*. After comparing the gene structures and motifs, we concluded the SlyHSF-25 and SlyHSF-26 that were located nearby one another on chromosome 2 had both lost Motif 4 and were duplicated from SlyHSF-16 (Fig. 3). These two duplicated genes may play a weak role in tomato metabolism activities due to much lower expression, both in all tissues and the heat treatment experiment.

## Supplemental Information

10.7717/peerj.1961/supp-1 Supplemental Information 1 Supplementary Tables

## Additional Information and Declarations

### Data Availability

The following information was supplied regarding data availability: The raw data has been supplied as Supplemental Information.
